# Novel psychoactive substances: What educators need to know

**DOI:** 10.1002/cpt.538

**Published:** 2016-11-29

**Authors:** ZR Patterson, MM Young, FJ Vaccarino

**Affiliations:** ^1^ Canadian Centre on Substance Abuse Ottawa Ontario Canada; ^2^ Department of Psychology Carleton University Ottawa Ontario Canada; ^3^ University of Guelph Guelph Ontario Canada

## Abstract

Novel psychoactive substances (NPS) are synthetic, psychoactive drugs that are generally not under international regulatory control. NPS are frequently sold as alternatives to classic “street drugs” such as ecstasy or LSD. However, little is known about their pharmacology and toxicity and they therefore pose unknown health risks. Further, risk for harms are elevated because users often do not know what they are taking, and therefore cannot predict dose, potency, or other potential properties.

## NOVEL PSYCHOACTIVE SUBSTANCES

As of 2015, the European Monitoring Centre for Drugs and Drug Addiction (EMCDDA) was monitoring 463 NPS, of which 98 were identified for the first time in 2015.^1^ Based on their parent compounds and mechanisms of action, NPS can be divided into seven distinct categories: synthetic cannabinoids, synthetic cathinones (also referred to as amphetamine‐type stimulants), novel synthetic opioids, empathogens, psychedelics, dissociatives, and depressants. Among the substances identified by the EMCDDA, synthetic cathinones and synthetic cannabinoids are the most widely available, and represent the greatest diversity of substances, accounting for over 40% of identified substances.^2^

Synthetic cathinones vary in their pharmacological actions, but are typically potent inhibitors to the dopamine and norepinephrine transporters (DAT, NET) and are classified based on their DAT/SERT inhibition ratio. All cathinones exhibit greater activation of the dopaminergic system compared to their amphetamine analogs, and are therefore thought to produce more stimulant‐type effects and show greater risk for dependence.^3^ In 2016 the National Institute on Drugs of Abuse reported that “Bath Salts,” a common term for synthetic cathinones in North America, have been mixed in stimulant drugs such as cocaine or methamphetamine, and have been used as a substitute for 3,4‐methylenedioxymethamphetamine (MDMA) in “molly” or “ecstasy.” Some of the more common synthetic cathinone's that have appeared in the media and elsewhere are methylenedioxypyrovalerone (MDPV), mephedrone (“plant food”), and alpha‐PVP (“Flakka”).

In contrast, synthetic cannabinoids (SCs) uniformly act as agonists to the cannabinoid‐1 receptor (CB_1_) and are thought to produce effects similar to tetrahydrocannabinol (THC). However, SCs have a higher affinity to CB_1_ and are associated with more severe psychosis, agitation, and more intense sympathomimetic effects. Unlike naturally occurring THC, most SCs do not contain cannabidiol, which has been shown to have anxiolytic and antipsychotic properties. As the result of a higher affinity for CB_1_, as well as the absence of cannabidiol, SCs are associated with more harm than marijuana. Furthermore, several metabolites of SC (e.g., JWH‐018 and its 4‐ and 5‐hydroxyindole metabolites) also have a high affinity for CB_1_, and are biologically active, thus prolonging the psychoactive and physiological effects of the parent compound.^4^ SCs are typically purchased online or via the black market, and are then dissolved in a solvent and sprayed on dried plant material so that the end product appears more natural. The sprayed plant material is then placed in small packets and branded with names such as “Spice,” or “K2,” and sold as “herbal incense” or “herbal smoking blends.” SC products are frequently labeled “not for human consumption,” which might be an attempt to circumvent drug laws in the jurisdictions in which they are sold.

Although synthetic cathinones and SCs account for a large number of the new substances identified, it is important to note that there are many other substances that have appeared in the recreational drug marketplace. Of particular note are the novel synthetic opioids, which have appeared in counterfeit pharmaceutical products and powders for sale in the illicit marketplace.

## CHALLENGES FOR EDUCATORS

The rate at which NPS appear poses a number of difficulties for those attempting to monitor the presence of, and harms associated with, these substances. Laboratories are struggling to keep up with appropriate analytical methods to identify new substances and governments/regulating bodies are attempting to develop new, or amend existing, legislation to control new chemicals. NPS also pose challenges for educators attempting to provide appropriate information to those who use these drugs. This is largely due to two factors: 1) there is little research assessing the pharmacology and toxicity of new substances, and 2) most people using these drugs do not know what or how much drug they are using.

### Limited information on NPS

For many NPS there is little research published because of the rapid life cycle of these substances—by the time appropriate pharmacological information is gathered and the harms are thoroughly and scientifically assessed, the substance has either disappeared from the market or has already caused significant harm.

Where there is published research, it is typically case studies with little statistical power or animal studies for which direct human comparisons are difficult. For many NPS there is simply no reliable information available. Those interested in learning more about the substance frequently look to user‐driven educational and harm‐reduction forums such as Erowid or bluelight.ru.

### People do not know what they are taking

People using drugs cannot be sure of the compounds they are consuming, and can be sold NPS, unknowingly, when seeking other “known” substances (e.g., “crack” or “ecstasy”). Further, the production and packaging of NPS occur in crude laboratories that lack quality assurance and testing. This leads to inconsistencies and variabilities in the quantity, purity, and potency of the active ingredient within and between batches of drugs.

## INTERVENTIONS AIMED AT PREVENTING OR REDUCING HARMS

### Prevention/education

Prevention and educational campaigns remain one of the most effective strategies for reducing NPS‐related harms. Effective prevention can be achieved by focusing resources on community needs and evidence‐informed practices, and by adopting a comprehensive approach by linking with other ongoing initiatives. The most common prevention programs currently focus on drugs such as cannabis, or target risk factors associated with substance use at large. These universal approaches are unlikely to be cost‐effective when it comes to preventing NPS use. Generic prevention programs should be adapted to include information on NPS specifically, and should be carefully monitored and evaluated to ensure its effectiveness. Evidence‐based resources such as the Portfolio of Canadian Standards for Youth Substance Abuse Prevention and the European Drug Prevention Quality Standards can be used in the development, or adaptation, of prevention programs aimed at NPS use. The Portfolio of Canadian Standards for Youth Substance Abuse Prevention is an evidence‐based resource that supports prevention teams on how to best plan, select, implement, and evaluate their prevention initiatives. These standards are designed to ensure a comprehensive approach to substance use prevention. The European Drug Prevention Quality Standards provide information on the core components of evidence‐based prevention interventions. Together, these resources guide the development, adaptation, evaluation, and refinement of effective prevention interventions.

### Drug checking

Drug checking and testing is a harm‐reduction strategy used to test the contents of pills and powders. The rationale for drug checking is that some people do not respond to prevention/education strategies and will use substances despite the associated risks. In these cases, it is argued that having information on the constituents of pills or powders purchased in the illicit market permits the user to make more informed decisions. The evidence to date suggests that drug checking can reduce harms associated with NPS use.^5^ However, it is important to highlight that the effectiveness of drug‐checking is limited to the validity and reliability of the methods used—some methods lack the sensitivity to detect some NPS and there is the possibility the results can incorrectly suggest safety. As with most harm‐reduction strategies, a key component is the opportunity for trained professionals to interact and educate people regarding the limitations of drug checking and the risks associated with substance use. Therefore, the value of drug checking is vastly diminished if not paired with appropriate education and messaging to highlight limitations, give context to test results, and deliver appropriate messages about substance use and ways to reduce harms. Standards and guidelines for drug checking should be developed to help improve the quality, consistency, and effectiveness of this approach.

### Treatment

Treatments for dependence on NPS follow the same strategies as common substance use disorder treatment programs. It is important to continue involving substance use services currently in place. The Novel Psychoactive Treatment UK Network (NEPTUNE) has developed a guidance document for the clinical management of harms associated with NPS. This document provides extensive and detailed information on harms associated with NPS use, and recommendations for the management of such harms. In collaboration with the Royal College of Psychiatrists, NEPTUNE is developing a series of e‐learning training modules for front‐line clinicians and other practitioners.

## CONCLUDING EDUCATIONAL CONSIDERATIONS

The uncertainty and unpredictability of the adverse effects associated with NPS use is perhaps the most important educational message. People often do not know what, or the potency of what, they are taking. Without emphasis on the uncertainty and unpredictability in our educational messaging, there is risk that people will equate the lack of information on NPS with reduced risk, rather than concern over the potential for unknown harms. Clinicians and researchers need to study the various NPS so that more information regarding their pharmacology and toxicity can inform interventions aimed at reducing harms. Preventative/harm‐reduction approaches should continue to educate people who use drugs about the possibility of not knowing what they are consuming, and the risks associated with polysubstance use, highlighting the full range of possible outcomes. Awareness of the presence of NPS and adulterants in common street drugs is extremely important to minimize harms. Treatment options and guidelines should follow those set in place for the parent compounds of NPS, although extra caution should be taken given the unknown and unpredictability surrounding NPS.

## CONFLICT OF INTEREST

The authors declare no conflicts of interest.
